# Effects of Group and Individual Culturally Adapted Cognitive Behavioral Therapy on Depression and Sexual Satisfaction among Perimenopausal Women

**DOI:** 10.3390/ijerph18147711

**Published:** 2021-07-20

**Authors:** Robab Khoshbooii, Siti Aishah Hassan, Neda Deylami, Rosediani Muhamad, Engku Mardiah Engku Kamarudin, Naser Abdulhafeeth Alareqe

**Affiliations:** 1 Department of Guidance and Counseling, Science and Research Branch, Islamic Azad University, Tehran 1477893855, Iran; r.khoshbooii@yahoo.com; 2 Department of Counselor Education and Counseling Psychology, Faculty of Educational Studies, Universiti Putra Malaysia (UPM), Serdang 43400, Malaysia; engkumardiah@upm.edu.my; 3 Department of Human Development and Family Studies, Faculty of Human Ecology, University Putra Malaysia (UPM), Serdang 43400, Malaysia; nedadeylamii@gmail.com; 4 Family Medicine Department, School of Medical Sciences, Health Campus, Universiti Sains Malaysia, Kubang Kerian 16150, Malaysia; rosesyam@usm.my; 5 Department of Educational Psychology and Counseling, Taiz University (TU), Taiz 6803, Yemen; nalareqe@yahoo.com

**Keywords:** CA-CBT, perimenopause, depression, sexual satisfaction, counseling

## Abstract

Aims: Previous research has shown the efficacy of culturally adapted Cognitive Behavioral Therapy (CA-CBT) in reducing depression, yet its effect on increasing sexual satisfaction is not well documented. In this study, an embedded randomized controlled trial design was used to examine the effect of group and individual CA-CBT on depression and sexual satisfaction among perimenopausal women. Method: A total of 64 depressed Iranian perimenopausal women were randomly assigned to two formats of treatments; sixteen sessions of group CA-CBT and eight sessions of individual CA-CBT, as well as a waitlist control group. Depression and sexual satisfaction were measured using BDI-II and ENRICH, respectively, at T1 (pre-treatment), T2 (post-treatment) and T3 (follow-up). Results: Repeated measures ANOVA indicated that the women who underwent both group and individual CA-CBT had effectively reduced depression and increased sexual satisfaction between pre-treatment and post-treatment, and it was sustained after six months of follow-ups with large effect sizes of significant differences (*p* < 0.001), but the control group did not. Conclusion: The results showed promising evidence for the efficacy of both treatment groups of CA-CBT for depression and sexual satisfaction among perimenopausal women. The population mental health burden among perimenopausal women may likely be reduced by propagating this effective treatment.

## 1. Introduction

Depression is a prevalent and impairing mental health disorder that affects women nearly twice more than men [1]. The high prevalence of depression has led to it being labeled as the “common cold” of mental disorders [2]. Depression has become a major societal concern because of the direct costs to healthcare and medical services, as well as the indirect costs of lowered productivity and disability [3]. Individuals suffering from depression are in a state of low mood, feel sad, empty and guilty, appear tearful, experience a loss of energy and interest in most activities, exhibit significantly decreased appetite or weight gain, and have insomnia or hypersomnia. They also avoid new jobs and situations due to feelings of inadequacy, worthlessness, aimlessness, and having suicidal thoughts [4].

Several significant risk factors have been identified that make women more susceptible to depression than men [1]. Studies reported that women are more vulnerable to depression during the developmental stages of their life, around puberty, before menstrual period, during pregnancy, after giving birth, and during menopausal transition. Especially during menopausal transition, women who are affected by depression typically experience irritability, mood swings, loss of interest or pleasure, fatigue, loss of energy, and sleep disturbances. Their marital relationship is negatively affected due to experiencing low levels of energy, self-imposed isolation, loss of interest in activities, being quiet, withdrawn, irritable, short-tempered, harsh, and aggressive [4].

Furthermore, substantial evidence suggests a decline in libido is strongly related to the depth of depression in women [5]. A depressed person experiences loss of interest in previously normally enjoyed activities and hence, is less likely to engage in sexual activities and achieve sexual satisfaction [6]. A study conducted by Frohlich and Meston [6] investigated the relationship between depression and sexual problems from data of 2159 undergraduate students aged 19–25 years old. It was found that the depressed students reported lesser vaginal lubrication, experience more pain and have difficulties in reaching orgasm. Hence, depression may affect sexual satisfaction regardless of age groups due to its symptoms [7,8]. To some women, they experience a brief episode of mild mood changes, but others will suffer from much more serious conditions that require active intervention and emotional support [9].

### 1.1. Menopausal Transition

**Figure 1:**
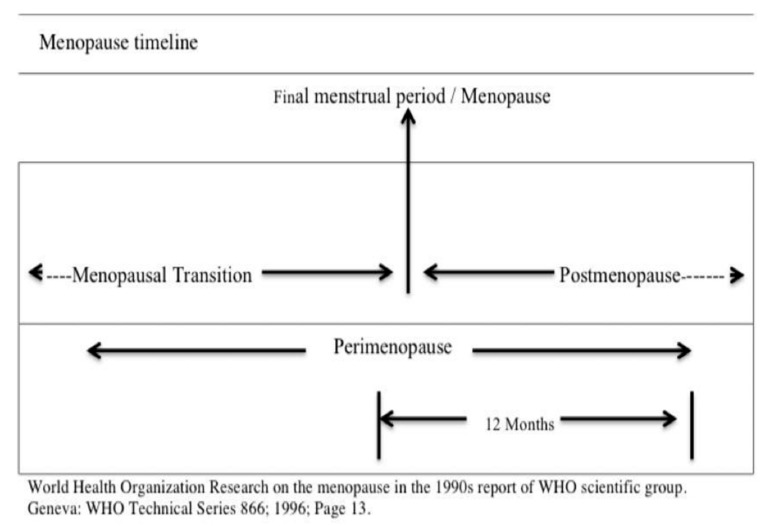
Menopause, perimenopause, and postmenopause.

Menopause is defined by the World Health Organization (WHO) as a permanent cessation of menses following twelve consecutive months of amenorrhea (the absence of menses). In other words, it is a point in time 12 months after a woman’s last menses. Figure 1 depicts menopausal transition and describes the states of menopause, perimenopause and postmenopause. Menopausal transition is a multidimensional process that contributes to the complex interactions between physiological and psychological factors, mostly between the ages of 45 and 55. The menopausal transition is also known as perimenopause, which usually lasts for about seven years, but can last as long as 14 years. On the other hand, postmenopause is when a woman has had no menses for an entire year and for the rest of her life after going through menopause.

Mohammad et al. [10] conducted a cumulative distribution study by region among 8194 women aged between 30 and 65+ who participated in this survey. The results showed that the mean age at menopause of the total population was 50.4 years (S.D. = 4.3). Another study conducted by Delaver and Hajiahmadi [11] reported that the median age of women in northern Iran who were approaching menopause was 48 years. Similarly, a study conducted by Abdollahi et al. [12] in Mazandaran Province in Iran indicated that the mean age of women who were approaching menopause in Mazandaran Province (Iran) is 47.93 years. Accordingly, by using the age of 50 as a proxy for menopause, it is estimated that approximately 47 million women will reach menopause per year, with 1.2 billion postmenopausal women living in the world by 2030, with an average age of about 60 [13].

### 1.2. Menopausal Transition and Sexual Satisfaction

Sex is among the main human motivations and sexual engagement is linked to better health and overall happiness [14]. Sexual satisfaction has the ability to produce reciprocal and shared pleasure in couples, as well as assist them in dealing more effectively with the tensions and troubles of everyday life. More diverse sexual activities for leisure have been reported during the COVID-19 pandemic due to movement restrictions [15]. A joyful sex life is a vital aspect in a happy and successful marriage, but a non-pleasurable sex life can lead to unhappiness and insecurity in couples [16].

Studies indicate that psychological factors play important roles in the sexual desire, activity, and satisfaction of women [2,17,18]. However, during the transition to menopause, women may experience changes in sexual functions due to several biopsychosocial risk factors, which can be identified in three major factors. First, women may lose sexual interests and desires as a result of psychological disorders, such as depression. Second, women’s sexual functions may suffer from menopausal symptoms, such as experiencing hot flashes, night sweats, and vaginal dryness due to a reduction in sex hormones as they get older, starting from 4 years prior to menopause [19,20]. These reduced hormones include estrogen and testosterone, which leads to the lessening of desires and receptivity for sexual activities, as well as their pleasures [19]. Third, cultural background, beliefs and attitude towards sexual satisfaction during middle age may also be significant factors in sexual behavior.

Regarding the first factor, women with depression may develop a negative view towards herself and her partner, who could be a part of a negative triad of depression [21]. Many researchers, like Beach et al. [22] reported that marital dissatisfaction is a function of depressive symptoms, communication and self-silencing in women. Yazdanpanahi et al. [23] indicated that depression affects sexual function. Bakhshi et al. [24] also found a negative relationship between depression and sexual satisfaction among Iranian couples. Khademi et al. [25] reported that women who suffered from mild to severe levels of depression exhibited sexual dysfunction. Thus, one of the major negative consequences of depression could be an increase of sexual dissatisfaction, which is assumed to be a factor for the increasing rate of divorce [26].

For the second factor, many studies have indicated that during the menopausal transition, women’s sexual problems are increased [27,28]. Javadivala, et al. [29] reported that 94.5% of participants in the postmenopausal stage experienced a decrease in sexual desire. Additionally, there is another research among Iranian women, in which it is reported that 70% of women after menopause had shown a reduction in sexual activities [30]. These findings reflected women approaching the menopausal age having a rapid decline in ovarian sex hormone levels, resulting in biological menopause and atrophic changes in the reproductive tracts [19]. Treating physical problems, such as vaginal dryness with moderate lubricant, is helpful and prevent women from experiencing painful intercourse [31]. However, providing physical treatment for these symptoms is not the only way to gain back normal responses to sexual intercourse. Female sexual response works dynamically, involving the interactions between both neurobiological and psychological dimensions [32]. Women need motivations to initiate and become receptive to sexual intercourse, which mainly include stability in their relationship, good emotions, pleasurable experiences in the previous sexual encounters and good mental health [33]. Many researchers explicitly highlighted that demotivation factors, such as depression and anxiety, were among undesirable viewpoints as it might reduce sexual desire and increase sexual dissatisfaction [33].

Finally, the third factor regarding the influence of cultural background on sexual interactions are influenced by historical roots, cultural beliefs, traditional attitudes, family structure, and philosophical values, thus, may influence sexual activity and desire among women [34], which in turn affects sexual satisfaction [35]. Abdolmanafi et al. [34] indicated that Iranian, had some common predictors of sexual dissatisfaction when compared to New Zealander women, such as thoughts of failure and disengagement, lack of erotic thoughts, and emotions of fear during sexual activity, as well as some specific cultural predictors, like sexual conservatism and women’s sexual passivity beliefs, and thoughts of sexual abuse.

### 1.3. Marital Sexual Satisfaction in Islam

Additionally, it is worthy to note that Iranians are influenced by both Islamic values and Iranian traditional culture; for example, sexuality in this culture is more related to procreation, while in Western societies, sexuality is more considered as recreational activity and sexual pleasure is expected. In Iranian families, discussion on sexuality is taboo and pre-marital sex is forbidden and many couples, particularly middle-aged women living with young adult children, are reluctant to have sexual interaction in their marital life. Also, limited expression of sexual desire is an indicator of purity, modesty. and a sense of reservedness, thus suppressing sexual desire during the menopausal transition is considered normal. This misconception of the ideal Muslim wife is characterized by sexual submission and modesty, and inhibition of sexual desires [33,36,37,38].

On the other hand, according to the Islamic transcendental wellbeing model, Hassan [39] explains that the spiritual dimension is the central dimension for Muslim women. Spiritual wellbeing permeates other dimensions of wellbeing, including the psychosexual dimension. In line with the study by Prause et al. [14] sex is positively associated with health and life satisfaction. Contrary to the popular belief and the misconceptions about ideal Muslim wives, achieving marital sexual satisfaction is highly encouraged among married Muslim women [37,39]. The sex manual is regarded as a sacred text that emphasized that sexual interactions between husbands and wives will absolve the sins of lovers.

Furthermore, in addition to the prescription of the Quranic verses and descriptions from Hadith (Prophet Muhammad’s tradition) on the romantic relationships between husbands and wives, extensive literature is available from pioneer Muslim scholars on how to help married women enjoy sexual interaction with their husbands [40]. Explicitly, in his book entitled *The Perfumed Garden of Sensual Delight*, Nafzawi [41] condemned men for ignoring their wives’ sexual satisfaction. “He inserts his flaccid little thing inside her only with the greatest difficulty, jerks once or twice then haul himself off her, taking longer to do so than he did on the job”. Additionally, Islam stresses the importance of foreplay, such as kissing, touching and the like, which are explicitly mentioned in Hadith. Sexual intercourse has to be avoided unless foreplay takes place, according to Sunnah. Prophet Muhammad forbids acting like an animal with one’s wife, which indicates sexual intercourse without foreplay [37].

### 1.4. CA-CBT Theoretical Framework

An amalgamation of al-Balkhi [42] and Beck’s [21] theories of Cognitive Behavioral Therapy (CBT) was used as the theoretical framework of this study. Since the Western theoretical framework on CBT and depression by Beck [17] is well known, this section focuses on Al-Balkhi’s theory of Cognitive Behavioral Therapy and methods for treating depression. Al-Balkhi was a Persian-born, Muslim pioneer of knowledge, and a physician who wrote about Cognitive Behavioral Therapy in his manuscript entitled “*Sustenance of the Soul*”, which was translated and annotated by Badri [42]. He was the first scholar in the 9th Century who was known for his astounding theory on CBT, and he differentiated between neurosis and psychosis and elaborated on psychosomatics [43].

Al-Balkhi described acute depression as a blazing coal fire, while sadness is analogous to the coal that remains glowing after the fire has subsided. The symptoms include an exhausted body, drained energy and a loss of desire in pleasurable activities. Sadness is the opposite of joyfulness and happiness. The face of a happy, elated person radiates with cheerfulness and brightness, while that of the depressed expresses gloom, pessimism and despair. Al-Balkhi theorized that fear and anxiety are caused by the expectation of a future threat and, on the other hand, sadness and depression are caused by the loss of something the person loves or is attached to. In short, fear is related to the future and sadness to the past. Individuals afflicted by both symptoms of fear and sadness experience the worst psychological symptoms, and have unhappy, miserable, and dejected lives.

Al-Balkhi further explained there are two types of depression and process of treatments. The first type has a clear identifiable cause, such as the death of a beloved person, the loss of wealth or something valuable to the person. The second type has no obvious reasons; it is a sudden distress and gloom affecting individuals, which prevents them, most of the time, from activities that are usually enjoyable and pleasurable. Al-Balkhi mainly focused on the treatment for the first type of depression, a normal reactive and endogenous one. For the second type, he briefly explained the treatment of physical and psychological treatments. He then suggested internal (cognitive) and external (behavioral) approaches for treatments for depression. The internal approach is the cognitive strategies, the optimistic thoughts that individuals cultivate within their souls to help them overcome any melancholic feelings that arise as a result of losing what they value or failing to achieve something that they crave.

Al-Balkhi’s cognitive strategies can be summarized as the following:

(1). To evaluate the physical harm that continuous melancholy and depression might inflict against the need to grieve over one’s loss. In anguish over what one has lost, destroying one’s health would be equivalent to someone selling up their capital for a small profit.
(2). To look around and realize that none in this world has perpetual enjoyment and happiness, none never loss of anything or anyone. The pleasures one acquires in life are merely an added gift to be enjoyed with delight, and the losses one experiences and the pleasures which one is unable to obtain should not stop one enjoying the present life with what one has.
(3). To train oneself to behave and endure in the face of misfortune by imagining the worse that could have happened, what will happen when one faces a greater calamity in the future if they cannot face it now.
(4). Modeling on the tales of courageous heroic people rather than succumbing to being cowardly and remain in sadness.
(5). Acknowledging that the painful occurrence and the days that follow will gradually diminish the incident’s terrible effects until it is forgotten. This mental strategy will produce an immediate feeling of comfort, if not outright delight and joy.

The behavioral strategies that are suggested by al-Balki are the following:

(1). Talking to someone who can bring back happiness.
(2). Listening to music and songs.
(3). Participating in activities that provide warm emotions.

### 1.5. Research Gap

As suggested by Azhar and Varma [44], an adaptation of CBT among the Muslim population is necessary for treatment to be effective. As in any other psychotherapy, CBT is value-laden, which involves the exploration of core beliefs and unhelpful patterns of thinking and attempts to modify them [42,45,46]. Hays and Iwamasa [47] suggested that religion and spirituality definitions, prejudice and political background, linguistic levels and cognitive style, family structure and gender role assignments, collectivistic orientations, and health belief perspectives are among the relevant culture-specific variables. According to Li et al. [48] it is important to consider cultural norms for the effectiveness of the intervention to maintain relevance to the intended population, in other words to be worthy of external validity.

Moreover, CBT, which is short-term, focused, cost-effective, and evidence-based, has been culturally adapted in different continents. In a recent review of meta-analyses of culturally adapted interventions, we found that CBT was the most commonly used culturally adapted therapy [46]. While there is a large empirical support for CBT as an effective intervention for treatment of depression [49], there is limited research on the effectiveness of CA-CBT for treatment of depression and sexual satisfaction among Iranian women in menopausal transition [46,50]. Considering the fact that culture has a fundamental influence on shaping attitudes, values systems, and points of view toward sex, it is essential to have a culturally adapted CBT as suggested by Hays and Iwamasa [47]. While the prevalence of depressive symptoms among perimenopausal women in Iran are high [51], there is a lack of study on a sample of perimenopausal Muslim women around the globe, and particularly in Iran.

To address this gap, we conducted the current research based on evidence of previous studies and hypothesized that the proposed intervention of culturally adapted Cognitive Behavioral Therapy (CA-CBT) would reduce depressive symptoms and concomitantly increase sexual satisfaction. In Iran, treatment protocols that included Quran verses for Muslim Iranian perimenopausal women who suffer from depression were based on Younesi et al. [52,53]. In Malaysia, CA-CBT is considered as a modification model of mainstream counseling practice that is adapted to be shariah-compliant [54]. For sexual satisfaction, the treatment protocol was adopted from Hassan [55].

### 1.6. Research Objectives

The objective of this study was to examine the effect of group and individual CA-CBT on depression and sexual satisfaction among perimenopausal women. Specifically, this study aimed to test the following hypotheses:

1. There is a significant reduction in depression and improvement in sexual satisfaction in the participants of the treatment groups GCA-CBT and ICA-CBT across time (T1, T2, and T3).
2. In comparison to the control group, the treatment groups remain significantly lower in depression and higher in sexual satisfaction at the follow-up time (T3).

## 2. Method

### 2.1. Research Design

**Figure 2:**
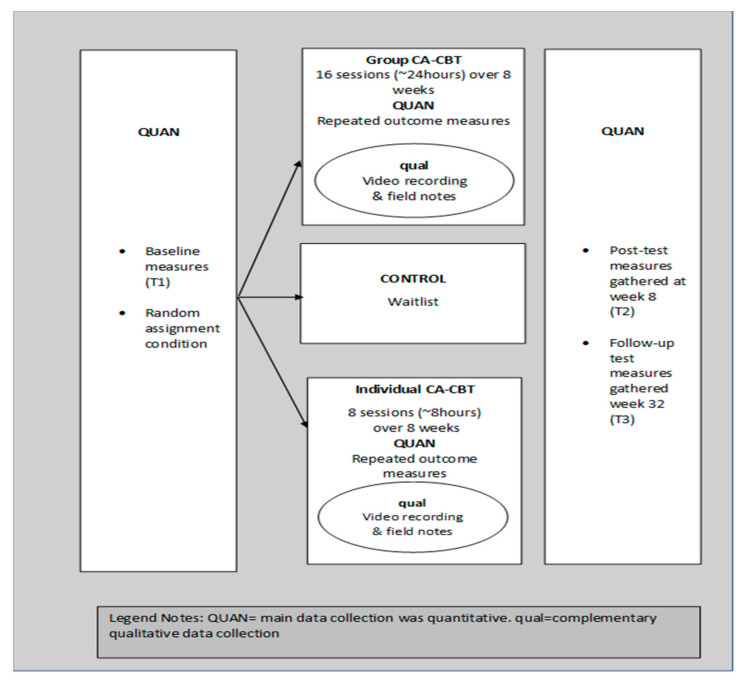
Research design of embedded randomized controlled trials.

This study employed an embedded randomized controlled trials design [56], which is a mixed method within the main quantitative strand [57]. Interpretive qualitative data analysis was used to enhance understanding of the randomized controlled trials (RCT) results [58] As shown in Figure 2, the main data collection was quantitative and complemented with qualitative data within the RCT. First, quantitative data were collected at pretest (T1) as the baseline data. No qualitative data were collected at T1. Similarly, no qualitative data but quantitative data were collected after the 8 weeks of treatment at posttest (T2) and at week 32 for follow-up test (T3). However, qualitative data were collected during the 8-week treatment. The qualitative data were collected to complement and enhance interpretation of the quantitative data of self-report measures.

**Figure 3:**
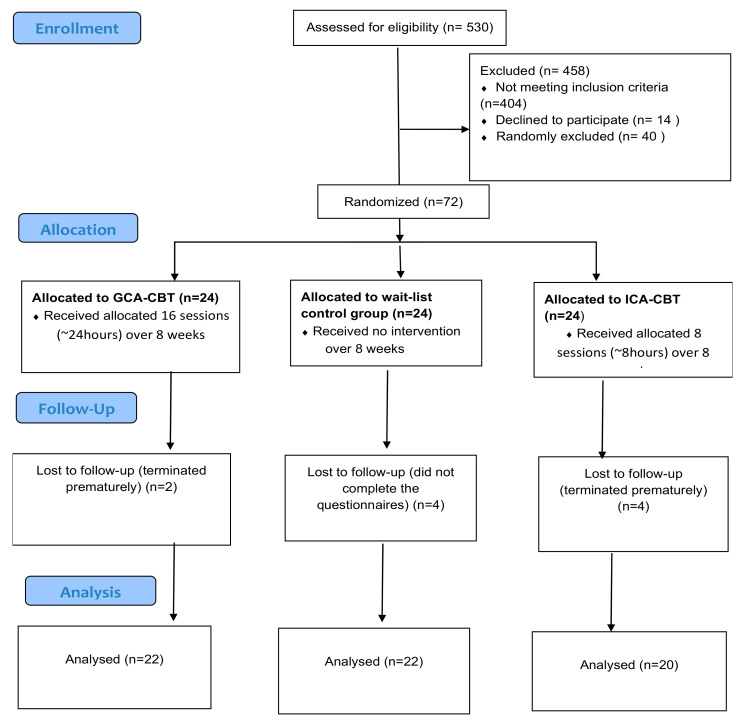
CONSORT Flow Chart of the randomized controlled trials (RCT) parallel arm for sampling procedure.

In this present study, the randomized controlled trials (RCT), followed the gold standard method according to Consolidated Standards of Reporting Trials (CONSORT) [59]. As shown in Figure 3, we adapted the CONSORT Flow Chart for the RCT parallel group trial design [60], a.k.a. multiple arm design [61], “It is ideal to include a zero-dose, or placebo, arm to avoid a situation in which all doses show similar activity and to establish whether any of the doses was superior to no treatment” [61]. In this study, we used subtypes of RCT consisting of two treatment arms, which are (1) the group culturally adapted cognitive behavioral therapy (GCA-CBT) and (2) individual culturally adapted cognitive behavioral therapy (ICA-CBT); and a control no treatment waitlist group. For ethical consideration, after the follow-up test (T3), we provided the waitlist control group with 8 sessions of GCA-CBT (~8 h) over 4 weeks.

### 2.2. Ethics Approval and Informed Consent

Ethical approval: All procedures performed in this study were in accordance with the ethical standards of The Ethics Committee for Research Involving Human Subjects Universiti Putra Malaysia (JKEUPM), which were guided in its stance and decisions by the principles expressed in the Declaration of Helsinki [62].

Informed consent: Informed consent was obtained from all participants included in this study. They were fully aware of various aspects of the research-the aim of the research, the procedures, and their right to withdraw from the study whenever they wished.

### 2.3. Participants and Setting

As shown in Figure 1, out of 530 women from four selected health centers in Tehran, Iran, 72 participants who met the criteria were randomly assigned to two treatment formats: individual and group CA-CBT, and a waitlist control group. The inclusion criteria were women with depression (BDI-II score of 21–46), aged between 40 and 55 years old who are in menopausal transition, with a minimum qualification of primary school education. The minimum qualification of primary school education was decided to ensure accessibility for more women. The exclusion criteria were women who are currently under medication or psychotherapy, have a previous experience of CBT, severe suicidal thoughts or psychotic depression and have a severe illness. From the 72 recruited participants, 2 and 4 participants in the treatment group and individual CA-CBT, respectively, refused to complete the sessions, and 4 participants in the control group were dropped. Finally, there were 64 participants who remained in this study for analysis.

### 2.4. Instruments

BDI-II. This 21-item self-report instrument is designed to assess the existence and the severity of depression symptomatology [63]. Each item was rated on a 4-point intensity, from 0 to 3, with 0 indicating no depressive symptomatology and 3 indicating a severe level of symptomatology. The total score ranged from 0 to 63 and the cutoff point was 17. This study used the translated and standardized version of BDI-II for an Iranian population. Ghasemzadeh et al. [64] examined the psychometrics of the Persian version of BDI and reported high internal consistency (alpha = 0.87) and an acceptable test-retest reliability r = 0.74 of the questionnaire. In this study, the Cronbach’s Alpha for the Iranian version of BDI-II at T1, T2, and T3 were 0.88, 0.94, and 0.94, respectively.

ENRICH subscales. The ENRICH Couple Inventory—Version, 2000 [65] was a 165-item multidimensional inventory that was a reliable and comprehensive assessment tool of the couple system. The ENRICH Couple Inventory contained 20 scales that were divided into four major groups: personality assessment, and interpersonal areas [65]. In this study, based on the research objective, the subscales of sexual satisfaction from interpersonal areas of the ENRICH Couple Inventory were selected. Therefore, the variable of sexual satisfaction was measured using the 10 items that are sexually related to the ENRICH questionnaire. It is a self-report measure of a couple’s sexual satisfaction and is used to measure problem areas in a couple’s relationships. Each item is rated based on a 5-point Likert-type scale agreement, with a rating from 1 indicating strong disagreement, to 5 indicating strong agreement. The total score of sexual satisfaction ranged from 10 to 50 points. Higher scores mean more satisfaction. The results from discriminant analysis ENRICH showed happily married couples could be discriminated from unhappily married couples, with 85–95% accuracy [66] being reported among Iranian [16]. The reliability of the Iranian version of this subscale was reported by Arab Alidousti et al. [67] with a Cronbach’s Alpha of 0.74. In this study, the Cronbach’s Alpha for the Iranian version of sexual satisfaction at T1, T2, and T3 were 0.70, 0.90, and 0.90 respectively. Demographic Questionnaire: Items in the questionnaire were about demographic characteristics that included age, educational level, number of children, income, job, physical health and history of depression. There were five items concerning the quality and regularity of monthly menstruation in the past six months to cover the menopausal status.

The instrument for qualitative data was the counselor (the first author) using counseling interviews. Data were obtained from verbatim transcripts of video recordings of the 16 sessions for the GCA-CBT and 8 sessions for the IGCA-CBT. The verbatim transcriptions and progress notes were scanned electronically after each session. They were coded by hand, and analyzed thematically, which enabled new insights and served as guidance for subsequent interviews [68]. The earlier transcripts were to be revisited throughout the process of coding and theme allocation over the 8-week treatments.

### 2.5. Procedure

Participants who were identified as perimenopausal women by gynecologist assessment were asked to confirm in writing their agreement to participate in the research. All participants were assessed three times: before treatment began (T1), at the end of the treatment on week 8 (T2), and during a follow-up session 6 months after the end of the treatment on week 32. Depression was diagnosed based on BDI-II and structural interviews. The participants were also recruited to answer the ENRICH questionnaire for their sexual satisfaction. The score of the pretest was considered as a baseline for the study outcome. Group and individual CA-CBT sessions were given to the participants of the treatment group over 16 two-hour sessions, which were carried out twice a week. The first author conducted all sessions with an assistant. The control group did not receive any intervention until the end of the study. The participants in the control group were offered group CBT upon completion of the study, including assessment procedures (pre-post-follow-up) as followed by the participants in the treatment group.

### 2.6. Treatment

As shown in Table 1, eight and 16 sessions of GCA-CBT and ICA-CBT treatment for depression and marital satisfaction for Iranian perimenopausal women were given, respectively. Firstly, the instructions for the treatment sessions were based on Al-Balkhi [42], Beck [21], and White and Freeman [69] on group Cognitive Behavioral Therapy (CBT). Therapy sessions covered the main component of CBT and culturally adapted components, which included: psychoeducation, cognitive interventions and behavioral interventions. For psychoeducation, the participants were enlightened on the many aspects of depression, and menopausal transition and they were familiarized with the cognitive model of depression. The subjects of automatic negative thoughts, cognitive distortions, dysfunctional assumptions, testing thoughts and beliefs were covered in the cognitive intervention stage.

Secondly, alternative thoughts were guided by the Quran verses in the cognitive intervention stage according to Younesi et al. [52,53]. The idea that our thoughts, beliefs, and behaviors all play a role in influencing our emotions is prevalent in the Quran and Hadith. God has firmly assured believers in the following verse: “*Your Lord has not abandoned you, nor does He despise you.*” (Quran, 94:5–6). “*We send the messengers only to give good news and to warn, so those who believe and mend (their lives)–upon them shall be no fear, nor shall they grieve*” (Quran, 6:48). *“Verily those who say, ‘Our Lord is Allah’, and remain firm (on that path, on them shall be no fear, nor shall they grieve*” (Quran, 46:13).

Thirdly, the subject of attitude, false belief and assumptions related to sexual satisfaction were discussed according to Hassan [39,45,55,70,71,72]. Behavioral intervention included modification of behavior, self-monitoring of daily behavior, monitoring mood and activities, rating mood related to activities, introducing the concepts of mastery and pleasure in activity and graded tasks. The behavioral intervention also covered social skills, like assertiveness and problem-solving strategies. Relaxation, and listening to and watching shariah-compliant songs and videos were also introduced. In her keynote speech at the International Conference on Culture, Psychopathology and Education in Tehran, Hassan [71] provided empirical evidence on the significance of love songs for marital counseling.

Fourthly, assigning homework to enhance the skills taught in the therapy sessions was a main component of the treatment. In this part, unrealistic thoughts and beliefs relating to their personal life stressors and sexual relationships were not directly challenged and disputed, rather possible solutions for alleviating depression were identified using Quran verses. We introduced Iqra bibliotherapy. “*Read in the name of thy Lord who created; [He] created the human being from blood clot. Read in the name of thy Lord who taught by the pen: [He] taught the human being what he did not know.*” (Quran, 96:1–5). We demonstrated how to search the relevant verses in the Quran, especially those related to sexual relationships, and we assigned them to search the verses as homework. Each session began with reviewing the given homework, setting the agenda in collaboration with group members, especially for GCA-CBT, followed by a short lecture, with the participants being assisted to apply CA-CBT skills regarding their personal problems.

### 2.7. Data Analysis

All statistics were analyzed using the Statistical Package of Social Sciences (SPSS) version 25. Descriptive statistics were used to analyze participants’ demographic information. Then, the assumption of normality of dependent variables and homogeneity of variance between the two groups were tested. For baseline results, the Levene’s test was used to show no significant difference in any of the three groups that indicated the homogeneity of groups at the pre-treatment time (T1).

In addition, Pearson’s product-moment correlation coefficient analysis was used to assess the relationship between depression and sexual satisfaction at pre-treatment time. The results indicated the collected data were deemed appropriate for a parametric test. An a priori power analysis according to Cohen’s [73] sampling table indicated a minimum n = 21 for each of the three groups of ANOVA design, with a power of 0.8 that showed a large effect size and a α value of 0.05.

The qualitative data were collected to complement and enhance interpretation of the quantitative data from the self-report measures. Transcripts were coded by hand, and thematic analysis was used to examine the transcripts. The use of thematic analysis enables new insights to inform the subject guide for subsequent interviews and earlier transcripts to be revisited throughout the process of coding and theme allocation [68].

## 3. Result

### 3.1. Demographic Characteristics and Baseline Result

The demographic characteristics of the participants were demonstrated in Table 2 as follows: The mean and standard deviation of the participants’ age were 48.77 and 4.54 years (from 41 to 55), respectively; 30% were of the age of 40–45, 34% were of the age of 46–50, and 36% were of the age of the 51–55. The breakdowns of educational qualifications were: 32% with primary school education, 55% with lower than high school education, and 13% higher than high school education. As for jobs, 68% were housewives, 14% currently employed, and 18% were retired. Economically, 36% of the participants had very low income, 41% had a low income and 23% had a moderate income. On the number of children: 27% have 0–2 children, 63% have 3–6 children, and 9% have more than 6 children. Overall, most of the participants belonged to the middle and low socio-economic class. It was discovered that there was a significant negative correlation between depression and sexual satisfaction r = −0.65, N = 44, *p* < 0.001 among participants of the study. The baseline results indicated that the following study on the effectiveness of CA-CBT on depression reduction and increment of sexual satisfaction was tenable.

### 3.2. Reduction of Depression and Increment of Sexual Satisfaction

As shown in Table 3, two tests within-subject repeated measure ANOVA for the treatment groups were conducted to compare pretest, posttest and follow-up scores on depression and sexual satisfaction across time.

The results illustrated that within treatment of GCA-CBT, there was a statistically significant decrease from pretest to posttest and follow-up on the depression scores F (1, 21) = 269.60, *p* = 0.001, ηp^2^ = 0.92, *f* = 3.2 which implied a large effect size [73]. The results also showed that there was a statistically significant increase in sexual satisfaction (F (1, 21) = 214.88, *p* = 0.001, ηp^2^ = 0.91, *f* = 2.9), which indicated a large effect size [73]. Post-hoc pairwise comparisons using the Bonferroni test indicated that the mean score for depression at T1 (M = 33.95, SD = 9.6) was significantly different from T3 (M = 12.63, SD = 6.41) and the mean scores for sexual satisfaction at T1 (M = 17.04, SD = 4.68) was significantly different from the T3 (M = 32.36, SD = 5.69).

For the ICA-CBT treatment, the findings indicated that there was a statistically significant difference in the depression scores between T1, T2, and T3 over time, (F (I,19) = 422.85, *p* = 0.001, ηp^2^ = 0.95, *f* = 4.24), which indicated a large effect size based on Cohen [54]. Post-hoc pairwise comparisons using the Bonferroni test exhibited a mean score for depression at T1 (M = 32.30, SD = 8.73). Results also showed significant differences from T3 (M = 11.75, SD = 6.59) and the mean scores for sexual satisfaction at T1 (M = 19.00, SD = 4.75) were significantly different from the T3 (M = 33.30, SD = 5.67) measurements. There was a statistically significant difference in the sexual satisfaction scores between T1, T2 and T3 over time (F (1, 19) = 118.77, *p* = 0.001, ηp^2^ = 0.86, *f* = 2.1), which indicated a large effect size [73]. Post-hoc comparisons using the Bonferroni test indicated that the mean score for sexual satisfaction at T1 (M = 19.00, SD = 4.75) was significantly different from T3 (M = 33.30, SD = 5.67). In summary, there were improvements of about 62% for GCA-CBT and 60% for ICA-CBT in comparison with the control group in reducing depression among Iranian perimenopausal women.

### 3.3. Sustainability of GCA-CBT and ICA-CBT Effects on Depression and Sexual Satisfaction

As shown in Table 4, a test of between-subjects effects of one-way ANOVA on depression and another one-way ANOVA for sexual satisfaction were conducted at T3 between the three groups. There were statistically significant differences between the three groups in terms of depression scores at T3, F (2, 61) = 73.90, *p* = 0.001, ηp^2^ = 0.70. *f* = 1.63). Post-hoc tests using the Bonferroni showed significant differences (*p* = <0.001) between group control group (M = 33.77, SD = 7.17), GCA-CBT (M = 12.63, SD = 6.41), and ICA-CBT (M = 11.75, SD = 6.59), but not statistically significant differences between both treatment groups. The mean differences between the control group and treatment group CA-CBT were 15.39. While the mean difference between the control group and individual CA-CBT group was 14.46. The magnitude effect sizes of the mean differences were *d* = 2.2 and *d* = 2.3, respectively, which were considered large. These results indicated that group CA-CBT and individual CA-CBT were effective in reducing and sustaining the level of depression among Iranian perimenopausal women.

The results also showed that there was a significant difference in the score of sexual satisfaction between the three groups at T3, (F (2, 61) = 56.57, *p* < 0.05, ηp^2^ = 0.65, *f* = 1.09), and a large effect size [73] was exhibited. Accordingly, a post-hoc pairwise comparison on sexual satisfaction showed statistically significant differences (*p* < 0.001). The mean differences between the control group (M = 17.90, SD = 4.57) and treatment group CA-CBT (M = 32.36, SD = 5.69) was 14.46, while the mean difference between the control and individual CA-CBT groups (M = 33.30, SD = 5.67). The magnitude effect sizes of the mean differences were d = 2.71 and d = 2.55, respectively, which were considered large. These results indicated that group CA-CBT and individual CA-CBT were effective in increasing and sustaining the level of sexual satisfaction among Iranian perimenopausal women. In summary, there were improvements of about 80% for GCA-CBT and 86% for ICA-CBT in comparison with the control group in increasing sexual satisfaction among Iranian perimenopausal women.

### 3.4. Complementing Qualitative Results on the Effects of ICA-CBT and GCA-CBT on Depression and Sexual Satisfaction

This study aimed to examine the effect of the treatment on depression and sexual satisfaction among Iranian perimenopausal women. Although marital sexual satisfaction seems to be a taboo topic, for the sake of research and academic advancement and with shariah-compliant treatments, we found that the participants became less embarrassed to share their experiences and were able to receive further support that they need to overcome their sexual dissatisfaction.

In the process of working with the treatment group, we discovered that participants tend to have negative thoughts about themselves, their marital relationship and the future of their marriage and children. Also, there were misconceptions regarding women’s role in sexual satisfaction, as well as myths and assumptions on menopause that influence women’s moods and behavior that have a significant influence on depressive symptoms. These false beliefs can lead to a cycle of self-criticism and hopelessness, and include feelings of guilt, excessive responsibility, and negative emotions during sexual activity. They often withdraw and avoid sexual relationships and feel worse and incompetent.

Some dysfunctional beliefs that were found during therapy sessions were: “Women cannot carry on with sexual activities after menopause”, “women do not have any desire for sexual satisfaction after approaching menopause”, “sexual function is necessary just for men”, and “women don’t need to be involved with this issue after the onset of menopause”. To help encourage the participants to share more information, a field note of the counselor (the first author) to neither directly condemn and dispute those beliefs, nor to preach them was always referred to as a reminder.

However, to help dispute the common theme of irrational beliefs, including “sex is dirty and sinful”; “experiencing pleasure during sexual activity is not acceptable in a virtuous woman”, we introduced and demonstrated, shariah-compliant Iqra bibliotherapy technique by searching the specific verse: “*If ye differ in anything among yourselves, refer it to Allah and His Messenger, if ye do believe in Allah and the Last Day: That is best, and most suitable for final determination*” (Quran 4:59). Then we explained that their belief about sex may not necessarily right, so it is best to refer the Quran, and try to understand what Quran says about sexual relationships.

Accordingly, we found that the participants seemed to be motivated with the given assignment and able to bring several verses of the Quran and they were discussed in the counseling sessions. Among the verses that the client brought for discussions were the following:

1. “It may be that you dislike a thing while it is good for you, and it may be that you love a thing while it is evil for you, and Allah knows, while you do not know”
2. “They (your wives) are your garment and you are a garment for them” [74],
3. “Among His signs is that He created for you spouses of your own kind in order that you may repose to them in tranquility and He instilled in your hearts love and affection for one another; verily, in these are signs for those who reflect (on the nature of the reality)
4. “Play any style and position you prefer”

In addition, the participants were also receptive to music and songs that were shariah-compliant as proposed by the respected Persian Muslim scholar Al-Balkhi. We demonstrated songs that are shariah compliance [39,54,55,70,71,72,75] Hassan [39,55] and provided evidence of the benefits. The participants were excited and one of them immediately shared some Persian songs and expressed that not only would she use the songs to strengthen her relationships with her husband, but she also will share this knowledge and experience listening to love songs and using the song lyrics to communicate with her newlywed daughter.

We also found that participants, particularly traditional and religious women, were not very open to discussing sexual interaction, sexual satisfaction, and problems in group CA-CBT treatment. However, in individual CA-CBT, the participants were less embarrassed and willing to share more of their problems and asked further questions.

## 4. Discussion

Generally, this study found that both treatments GCA-CBT and ICA-CBT were effective in treating depression and sexual satisfaction among perimenopausal women. The findings revealed that participants’ depressive symptoms were significantly reduced after 16 sessions of GCA-CBT and eight sessions of ICA-CBT. The comparison between the two treatment groups and the control group indicated that participants of the treatment groups reported a more statistically significant decrease in depression from pretest to posttest and follow-up than the control group. The findings in the group CA-CBT showed a large effect size (*f* = 2.9) and the findings in the individual CA-CBT also showed a large effect size (*f* = 2.1) and over time, it supported the practical significance of CA-CBT and its therapeutic value for depression. GCA-CBT was 62% more effective compared with the control group with no treatment in reducing depression based on the outcome of BDI-II scores with a large Cohen’s *d*-effect size of 0.92 among Iranian women at the menopause period. ICA-CBT behavioral therapy was 60% more effective compared with the control group with no treatment in reducing depression based on the outcome of BDI-II scores with a large Cohen’s *d*-effect size of 0.95 among Iranian women at the menopause period. The findings were in line with Jalal et al. [76] which provided evidence of the effectiveness of CA-CBT among the Muslim population in Egypt, and in line with Zakaria and Akhir [75] for the Malaysian population. Also, these findings are consistent with that of prior studies that suggested depression will often improve following CA-CBT intervention [5,76,77,78,79,80].

Moreover, the findings in the GCA-CBT showed a large effect size (*f* = 3.2) and the findings in the ICA-CBT also showed a large effect size (*f* = 4.2) and over time, supported the practical significance of CA-CBTs and their therapeutic value for sexual satisfaction. It supported the practical significance of CA-CBT and its therapeutic value for sexual satisfaction. GCA-CBT was 80% more effective compared with the control group with no treatment in increasing sexual satisfaction based on the outcome of ENRICH scores among Iranian women at the menopause period. ICA-CBT was 86% more effective compared with the control group with no treatment in increasing sexual satisfaction. The findings supported the effectiveness of CA-CBT on sexual functions and depression among women [39,81]. In tandem with Hays and Iwamasa [47], self-monitoring, of thoughts and feelings, as well as rating their mood related to different actions and behaviors helped the participants to understand their own problems. As suggested by Barzoki et al. [82], when one of the sexual partners are involved in satisfaction and orgasm, as only 8.5% of women have experienced orgasm in their entire sexual life, their displeasure and dissatisfaction eventually affects their men. This is because sexual satisfaction is a reciprocal interaction, where both partners can influence and be influenced by one another [37,39].

Hence, this study provides evidence on how to help Iranian women achieve sexual satisfaction by providing the appropriate information and train them with the effective skills to reduce their depression and misconceptions about sexual relationships. The findings extended prior research by demonstrating the effect of CA-CBT on depression, which was approved by Gilliam et al. [78] and sexual satisfaction, in line with Rajabi et al. [83]. Thus, cognitive distortion as the component of false beliefs and assumptions was distinctly emphasized. Apart from that, it was found that in addition to depressive symptoms, women’s sexual functions were largely influenced by a lack of social skills, such as assertiveness, and problem-solving appraisal, which could affect the severity of depression, which is in line with the study by Peleg-Sagy and Shahar [84] and Green et al. [79]. The treatment protocol was adjusted to meet the participants’ needs as a Muslim woman, as in the study conducted by Zakaria and Akhir [75]. These needs were the central dimension of wellbeing that encompasses the spiritual dimension and sexual dimension and were interrelated with other dimensions of wellbeing [39]. This is also in line with the study by Zabor et al. [61] in which sexuality among women should be viewed more comprehensively, as 79% of women complaining about sexual-related distress may not need medicine. The findings of this study extended prior research by demonstrating the effect of CA-CBT on depression that was approved by Gilliam et al. [78] and sexual satisfaction in line with Rajabi et al. [83] through a randomized controlled trial design.

### 4.1. Strength and Limitations

An important strength of this study is that we compared the effects of two active treatment conditions with no treatment condition in treating perimenopausal women for depression, and sexual satisfaction using an embedded randomized controlled trials with a parallel arm design as suggested by Zabor et al. [61], Tripolt et al. [56] and Creswell and Plano Clark [57]. To date, our study is the first of its kind to provide evidence of the effectiveness of CA-CBT with a large magnitude effect size for both treatment conditions. There was a 60% improvement for groups and 62% improvement for individuals for depression, and an 80% improvement for groups and 86% improvement for individuals for sexual satisfaction with CA-CBT. The qualitative findings of this study help enlighten the effectiveness and the acceptance of the treatment by the participants. Another worthy contribution is that this is the first study tested on the applicability and effectiveness of Al-Balkhi’s theory of CBT in combination with Beck’s theory, while others were describing Al-Balkhi’s theory [43]. However, there is a limitation to this study, as we did not have an active control group of treatment as usual (TAU) to control the Hawthorne effect on the waitlist control group [85]. Another limitation of this study is that sexual satisfaction is a mutual spousal relationship, but we only evaluated the wife’s sexual satisfaction [86].

### 4.2. Implication

The findings of this study have implications on therapists who work on sexual problems in women, and they should consider if indeed there is depression prior to the advent of sexual dysfunction. Depressed individuals often complain about low or no sexual desire, which puts a tremendous strain on close relationships with their partners. The results of this research study illustrate those sexual relationships are influenced by psychological disorders, notably depression during menopausal transition.

Practically, it is implied that cultural and social contexts have shaped women’s attitude and beliefs on sexual intimacy and relationships, especially for perimenopausal women. Contrary to studies that reported women were very open in discussing their sexual problems and talking about personal experiences [87], the findings of this study demonstrated that Iranian women, particularly traditional and religious women, were not very open to discuss sexual interaction, sexual satisfaction and problems. They were more comfortable discussing sexual issues privately in individual counseling sessions. This helps explain how the individual CA-CBT treatment exhibited an 86% improvement and the group CA-CBT an 80% improvement. Nevertheless, the difference was not significant, but significant in comparison to the no-treatment waitlist control group.

These difficulties of sharing sexual-related problems depend on either experiencing shame and shyness when talking about sex as a cultural taboo, and being difficult to consult with parents, and it even depends on the unknown experience of orgasm among women. Barzoki et al. [82] has indicated that only 5.8% of Iranian women have reported experiencing orgasms throughout their marital life.

For future studies, we suggest the extension of benefits from CA- CBT on depression and sexual satisfaction on a more ethnically diverse population from other states in Iran, because Iran has diverse ethnics groups, in which each of them have their own norms and values. Furthermore, a cross-national study involving different ethnicities to evaluate the effectiveness of CA-CBT on improving sexual satisfaction and decreasing depression signs among other perimenopausal women is recommended. These suggestions would help increase the generalization of the findings.

## 5. Conclusions

This study provides promising evidence, supporting the remarkable benefits of CA-CBT that is amalgamated with Al-Balkhi’s and Beck’s theories for treating depression and sexual dissatisfaction among perimenopausal women in Iran through embedded randomized controlled trial design. The effectiveness of the CA-CBT on depression and sexual satisfaction among Iranian perimenopausal women supported the applicability and flexibility of this intervention for a wide range of the population. Treatments that focused on women’s attitudes and dysfunctional beliefs about sexual relationships were indirectly challenged with homework assignments on searching the Quranic verses related to sexual relationships that seem to support the shariah-compliant model of treatment with receptive attitudes of the participants.

## Figures and Tables

**Table 1:** Summary for treatment of culturally adapted Cognitive Behavioral Therapy (CA-CBT) on depression and marital sexual satisfaction for Iranian perimenopause women.

Session	Purpose	Agenda	Homework
1–2	Psychoeducation Educate about Depression Socialize to CBT Motivate the patient	What is depression? Review symptoms of depression, Introduce the CBT approach to depression, explain cognitive and behavioral components of depression, explain the goal of therapy, eliciting goals from groups, and make a goal list.	What are the symptoms of depression? What are your symptoms? Make a problem list with personal priority
3–4	Activating the patient behavioral modification How activity affects mood. Modifying activities to improve mood.	Outline the relationship between mood and activities. Activity scheduling. Identify pleasurable and mastery activities. Specifying behavioral change to meet goals. Behavioral modification.	Keep a diary and enter activities; monitoring/rating mood and activity. What activities improve mood/worsen it?
5–6	Cognitive intervention: relation between situation, thinking, and mood; Identify negative automatic thoughts (NAT)	What are thoughts? What is self-talk? How can thought affect body, actions and mood? Introduce negative thoughts, negative automatic thoughts. NATs related to menopause and its symptoms	Three columns of DTR identify situation, thoughts and moods, identify your menopausal symptoms.
7–12	Testing NATs Identify cognitive distortion, underlie NATs	Testing NATs by group’s priorities, interpersonal conflicts, cognitive distortion; Explain dysfunctional rules and assumptions related to sexual satisfaction. Introduce Downward arrow	Complete DTR with alternatives, Cognitive distortions related to menopause and sexual satisfaction.
13–15	Problem-solving Introduce core beliefs	Problem-solving strategies. Discussion of core-beliefs based on Quran verses.	Problem-solving tasks. Identify “Rules of your mind”
16	Relapse prevention termination	Review of the therapy. Relapse prevention. Preparation for ending therapy	Applying the techniques in their lives.

**Table 2:** Sample characteristics of the three groups.

Characteristic	Control	CA-CBT	CA-CBT	Levene
	n = 22	(Group)	(Individual)	Test
		n = 22	n = 20	Sig.p
Age	Frequency (%)	Frequency (%)	Frequency (%)	N.A.
40–45	6 (28%)	7(32%)	5(24)	
46–50	8(36%)	7(32%)	7(33)	
51–55	8(36%)	8(36%)	8(40)	
Education				N.A.
Primary school	8(30%)	7(32%)	7(30%)	
Diploma	12(60%)	12(55%)	11(60%)	
Bachelor	2(10%)	3(13%)	2(10%)	
Children				N.A.
0–2	6(27%)	6(27%)	7(30%)	
3–6	14(64%)	14 (64%)	11(60%)	
6>	2(9%)	2(9%)	2(10%)	
Employment				N.A.
Housewife	15(68%)	15(68%)	14(70%)	
Employee	3(14%)	3(14%)	3 (15%)	
Retired	4(18%)	4(18%)	3(15%)	
Income				N.A.
>300	8(36%)	8(36%)	7(30%)	
300–599	9(41%)	9(41%)	9 (45%)	
600>	5(23%)	5(23%)	4 (20%)	
Income	M = 604,545	M = 598,181	M = 575,000	*p* = 0.92
	−2.27	−2.09	−1.81	
Age	M = 48.63(4.63)	M = 48.90(4.56)	M = 45.40(4.22)	*p* = 0.84
Sexual satisfaction	M = 17.86 (4.85)	M = 17.04 (4.68)	M = 19.00(4.75)	*p* = 0.57
Depression pre-test	M = 33.95	M = 34.09	M = 32.30	*p* = 0.96
	−9.6	−8.34	−8.73	

Note: N.A = not applicable.

**Table 3:** Repeated Measures ANOVA for T1, T2, and T3 for GCA-CBT and ICA-CBT.

Tests of within-Subjects on Depression and Sexual Satisfaction over Time in GCA-CBT and ICA-CBT											
		Depression				Sexual Satisfaction					
Group	Tests		Mean	SE	ρ-Value	Mean	SE	ρ-Value			
**GCA-CBT**	T1	T2	21.90 *	1.19	0.001 *	−16.09 *	0.98	0.001 *			
	T1	T3	21.31 *	1.35	0.001 *	−15.31 *	1.06	0.001 *			
	T2	T3	−0.59	0.45	0.622	0.77	0.45	0.304			
**ICA-CBT**	T1	T2	21.45 *	0.84	0.001 *	−15.55 *	1.32	0.001 *			
	T1	T3	0.55 *	1.04	0.001 *	−14.30 *	1.24	0.001 *			
	T2	T3	−0.90	0.53	0.331	1.25	0.69	0.260			
**Pairwise Comparisons of Three Times on Depression and Sexual Satisfaction in ICA-CBT and GCA-CBT**											
		**Depression**					**Sexual Satisfaction**				
**Group**	**Tests**	**Mean (SD)**	**df**	**F**	**ρ-Value**	**ηp^2^**	**Mean (SD)**	**df**	**F**	**ρ-Value**	**ηp^2^**
**GCA-CBT**	T1	33.95 (9.64)	1.21	269.60	0.001 *	0.92	17.04 (4.68)	1.21	214.88	0.001 *	0.91
	T2	12.04 (5.89)					33.13 (5.56)				
	T3	12.63 (6.41)					32.36 (5.69)				
**ICA-CBT**	T1	32.30 (8.73)	1.19	422.85	0.001 *	0.95	19.00 (4.75)	1.19	118.77	0.001 *	0.86
	T2	10.85 (6.17)					34.55 (5.76)				
	T3	11.75 (6.59)					33.30 (5.67)				

Note: * Sig. *p* < 0.01.

**Table 4:** One-Way ANOVA for GCA-CBT, ICA-CBT, and C-CBT at follow-up.

Tests of Between-Subjects Effects on Depression and Sexual Satisfaction at T3 among Three Groups								
	Depression				Sexual Satisfaction			
Source	Mean (SD)	F	ρ-Value	ηp^2^	Mean (SD)	F	ρ-Value	ηp^2^
GCA-CBT	12.63 (6.41)	73.90	0.001 *	0.70	32.36 (5.69)	56.57	0.001 *	0.65
ICA-CBT	11.75 (6.59)				33.30 (5.67)			
C-CBT	33.77 (7.17)				17.90 (4.57)			
**Pairwise Comparisons of Three Groups on Depression and Sexual Satisfaction at T3**								
**Group**		**Variables**						
		**Depression**			**Sexual Satisfaction**			
		**Mean**	**SE**	**ρ-Value**	**Mean**	**SE**	**ρ-Value**	
GCA-CBT	ICA-CBT	0.88	2.08	1.00	−0.93	1.64	1.00	
	C-CBT	−21.13 *	2.03	0.001 *	14.46 *	1.60	0.001 *	
ICA-CBT	C-CBT	−22.02 *	2.08	0.001 *	15.39 *	1.64	0.001 *	

Note: Adjustment for multiple comparisons: Bonferroni. * Sig. *p* < 0.01.
